# Ureido Hyperbranched Polymer Modified Urea-Formaldehyde Resin as High-Performance Particleboard Adhesive

**DOI:** 10.3390/ma16114021

**Published:** 2023-05-27

**Authors:** Hongxing Yang, Hao Wang, Guanben Du, Kelu Ni, Yingchen Wu, Hang Su, Wei Gao, Xiaoping Tan, Zhaojin Yang, Long Yang, Xin Ran

**Affiliations:** 1 Yunnan Province Key Lab of Wood Adhesives and Glued Products, International Joint Research Center for Biomass Materials, Southwest Forestry University, Kunming 650224, China; hongxing0778@126.com (H.Y.); wanghao961227@163.com (H.W.); nkl592664163@sina.com (K.N.); 18636323236@163.com (Y.W.); woodneedsaysh@163.com (H.S.); weigaoe@gmail.com (W.G.); yangzhaojin6@163.com (Z.Y.); long133109070@126.com (L.Y.); 2 Key Laboratory for Forest Resources Conservation and Utilization in the Sou thwest Mountains, Ministry of Education, Southwest Forestry University, Kunming 650224, China; 3 Kunming Feilin Panel Board Co., Ltd., Kunming 650224, China

**Keywords:** particleboard, hyperbranched modification, water resistance, formaldehyde emission, cross-linking network

## Abstract

The performance of urea-formaldehyde (UF) resin and its formaldehyde emission is a natural contradiction. High molar ratio UF resin performance is very good, but its formaldehyde release is high; low molar ratio UF resin formaldehyde release is reduced, but the resin itself performance becomes very bad. In order to solve this traditional problem, an excellent strategy of UF resin modified by hyperbranched polyurea is proposed. In this work, hyperbranched polyurea (UPA_6N_) is first synthesized by a simple method without any solvent. UPA_6N_ is then added into industrial UF resin in different proportions as additives to manufacture particleboard and test its related properties. UF resin with a low molar ratio has a crystalline lamellar structure, and UF-UPA_6N_ resin has an amorphous structure and rough surface. The results show that internal bonding strength increased by 58.5%, modulus of rupture increased by 24.4%, 24 h thickness swelling rate (%) decreased by 54.4%, and formaldehyde emission decreased by 34.6% compared with the unmodified UF particleboard. This may be ascribed to the polycondensation between UF and UPA_6N_, while UF-UPA_6N_ resin forms more dense three-dimensional network structures. Finally, the application of UF-UPA_6N_ resin adhesives to bond particleboard significantly improves the adhesive strength and water resistance and reduces formaldehyde emission, suggesting that the adhesive can be used as a green and eco-friendly adhesive resource for the wood industry.

## 1. Introduction

As the world’s population grows rapidly, the interaction between humans and nature has increased. This situation has accelerated the demand for forest products, particularly affordable wood composite boards such as particleboards (PBs) [1,2]. Moreover, particleboard manufacturers are striving to reduce the cost of production. In addition to wood costs, adhesives used in the production of PBs are the most significant cost factor. Urea-formaldehyde (UF) resins are the most commonly used resins, widely used for manufacturing PBs and other wood-based products [3,4]. UF resins were first synthesized in 1884 with the advantages of high reactivity, low cost, low cure temperature, high bonding strength, a colorless glue line, and so forth. Unfortunately, the major disadvantages of UF adhesives are free formaldehyde emission from them bonded wood-based panels and poor durability under high humidity conditions, especially when combined with heat [5,6]. Hence, the modification of UF adhesives is a critical research topic.

The rate and extent of formaldehyde release from PBs are influenced by various parameters, such as the mole ratio of F/U, catalyst level and composition, resin level, and moisture content, among which the F/U mole ratio is recognized as the most important factors [7,8,9,10]. It has been demonstrated that excess urea could remove the free formaldehyde, whereas the obvious adverse effect is that the branched hydroxymethyl chain on the polymer is also cleaved, leading to the formation of linear polymers. In order to reduce the amount of formaldehyde released, UF resin (usually ≤ 1.0) is synthesized by a low F/U molar ratio method in industrial production, and formaldehyde is still released continuously when used in wood products [11,12]. Furthermore, Park et al. reported that the formation of cross-linked structures of UF resins with low-molar ratios was suppressed due to the hydrogen bonding interaction between linear polymers [13,14]. It is worth mentioning that this is also the primary reason for the poor property of UF resin, especially for the high formaldehyde emission and poor water resistance. Therefore, modifying UF resin to improve properties and reduce formaldehyde emission is still a good option until an alternative emerges. Many modification methods have been attempted, and the most successful one is to prepare melamine-UF co-condensed resin (MUF) by adding melamine to UF resin [15,16,17,18]. The amount of melamine added determines its properties. In actual manufacturing, when the molar ratio of M/UF is greater than 1:1 or even higher. MUF exhibits mechanical properties similar to MF. At the same time, too high melamine content will lead to a significant increase in cost and storage stability.

In recent years, the hyperbranched structures have not only covered the “classical” products based on the AB_x_ approach but also extended to various highly branched structures, including those prepared by the A_2_ + B_y_ monomer combination, those prepared by self-condensing vinyl polymerization, ring-opening multibranching polymerization or self-condensing ring-opening polymerization, or proton transfer polymerization [19]. To take it one step further, the UF and MUF resins can be modified with linear or hyperbranched polymers [20]. In particular, poly(amidoamine) modifications with distinct terminal groups (including -OH, -NH_2_, -CHO, e.g.) are extensively studied in reported works [21,22,23]. Compared to linear polymers, highly branched polymers have unique topological structures and physical properties, such as better solubility, high toughness, low viscosity, and many end groups, which have attracted much attention. Regulating the three-dimensional (3D) structure of highly branched polymers (HBPs), i.e., molecular weight, dispersity, number of branching points, branching density, and chain-end functionalities, would significantly improve and modify the polymer properties and contribute to the design and synthesis of new polymer materials [24,25]. These superiorities make branched polymers promising in many applications, such as lubricants, coatings, antimicrobial agents, adhesives, and adsorbents [26,27,28]. Inspired by UF resin modification research, as a thermosetting wood adhesive, the hyperbranched cross-linking structure should be one of the most important factors in its adhesive property on account of the cross-linked density of the cured adhesive network can obviously be improved by high functionality and high branching degree, and the selective conversion of the live polymer end enables the introduction of amine functional groups at the chain end, which provides the possibility for the preparation of modified urea–formaldehyde resin based on HBPs [19,29]. From this, this work aims to explore the improvement and advancement of the UF adhesive by an ureido highly branched polymer reported by our group recently, which is named UPA_6N_ [30].

Hence, this study attempts to improve the performance of the low molar ratio UF adhesive using the UPA_6N_ ureido polymer. We used solvent-free and catalyst-free methods to synthesize UPA_6N_ by deaminating polycondensation reaction of urea and polyamine (PA_6N_) and added industrial gas UF and UPA_6N_ in different proportions as additives to obtain various UF-UPA_6N_ resin binders. This study focuses on the modification mechanism and macroscopic mechanical properties of UPA_6N_ resin on urea–formaldehyde resin with a low molar ratio and scale-up its performance testing through the wood-based panel continuous flat press production line in Yunnan XinZeXing wood-based panel Co., Ltd. In addition, the crystallinity, thermal behavior, chemical composition, and mechanical performance of the synthesized samples are determined by Infrared spectroscopy (FTIR-Thermo Scientific Nicolet iS50, Waltham, MA, USA), X-ray diffraction (XRD-Instrument: TTRIII-18KW Xray diffractometer, Rigaku, JP), Atomic force microscopy (AFM-BKG, Rhabdos Co. Ltd., Seoul, Korea), Differential scanning calorimetry (DSC-Perkin-Elmer DSC 7, Germany), and Transmission electron microscopy (TEM-JEOL JEM-F200, Germany) and so forth.

## 2. Experimental Sections

### 2.1. Chemicals and Materials

The triethylenetetramine, ethylenediamine, methyl acrylate, CH_3_OH, and urea were purchased from Titan Scientific Co., Ltd. (Shanghai, China). UF resin was provided by Yunnan XinZeXing wood-based panel Co., Ltd. (Kunming, China). It should be noted that the formula of the UF resin with molar ratio (F/U) of 1.03 was used in this study.

### 2.2. Synthesis of UPA_6N_ Ureido Polymer

**Figure 1:**
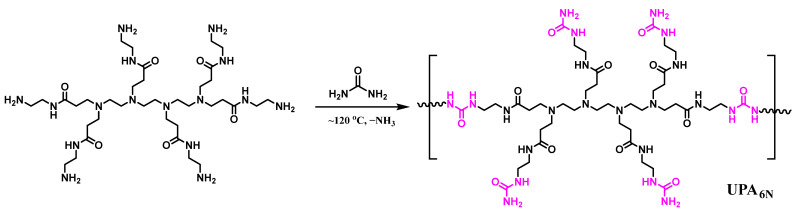
The synthesis route of UPA_6N_ via deamination polycondensation.

The UPA_6N_ was synthesized according to previously reported methods [30,31]. Briefly, PA_6N_ was synthesized by using triethylenetetramine and methyl acrylate via Michael addition reaction, and the products then reacted with ethylenediamine through nucleophilic substitution to obtain PA_6N_. The UPA_6N_ was then prepared by the deamination polycondensation between PA_6N_ and urea, as provided in Figure 1.

### 2.3. Preparation of UPA_6N_ Modified UF Resins

**Figure 2:**
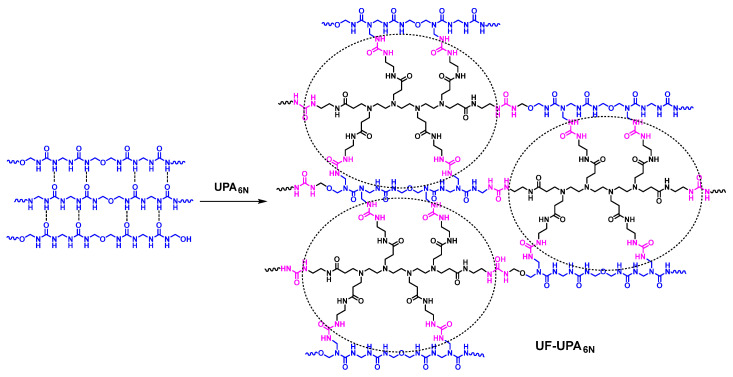
The proposed structural modification strategy of UF-UPA_6N_.

UF resin (F/U molar ratio 1.03) used in this study was provided by Yunnan XinZeXing wood-based panel Co., Ltd. Then, 10 kg UPA_6N_ (50 wt% solid content) aqueous solution was added into 1000 kg UF resin, named UF-UPA_6N_ (0.5%). UF-UPA_6N_ (0.25%), UF-UPA_6N_ (1.0%), and UF-UPA_6N_ (1.5%) were also prepared by adding 5 kg, 20 kg, and 30 kg UPA_6N_ into 1000 kg UF resin, respectively, as provided in Figure 2.

### 2.4. Characterization Methods

The obtained samples were tested by Fourier transform infrared (FTIR-Thermo Scientific Nicolet iS50, Waltham, MA, USA); X-ray photoelectron spectroscopy (XPS- XPS, ESCALAB 250Xi, Thermo Fisher, USA); Differential scanning calorimetry (DSC-Perkin-Elmer DSC 7, Germany) and Thermogravimetric analysis (TGA Instruments Q50, Germany); Transmission Electron Microscopy (TEM-JEOL JEM-F200, Germany); Atomic force microscopy (AFM-BKG, Rhabdos Co. Ltd., Seoul, Korea). UF resin and UF-UPA_6N_ resin were prepared with (0.5%) and without (0%) UPA_6N_, respectively, before observation and dried in an oven at 120 °C for 2 h. For the DSC tests, samples were weighed into an aluminum DSC pan and placed into the sample holder without a lid, where the samples were held isothermally at 20 °C under a 20 mL min^−1^ flow of nitrogen. In addition, for TGA analysis, all samples were held isothermally at 30 °C for 5 min and then heated from 30 to 800 °C at 10 °C min^−1^.

### 2.5. Preparation of Particleboard

Particleboard is made of eucalyptus wood shavings, of which the moisture content is 10–12%. The particleboard is prepared with the fiber of length 0.8–3.0 mm as the upper and lower surface layers and the large pieces of shavings with the specifications of (20–100) mm × (10–50) mm × (0.2–2.0) mm as the core layer. The original UF, UF-UPA_6N_ (0.25%), UF-UPA_6N_ (0.5%), UF-UPA_6N_ (1.0%), and UF-UPA_6N_ (1.5%) resin adhesives were used for particleboard production on the wood-based panel continuous flat press production line in Yunnan XinZeXing wood-based panel Co., Ltd. The prepared particleboard was cooled down to room temperature for 24 h to relieve internal stress. Then, each particleboard was cut into specimens with an area of 60 cm × 60 cm for the following tests. The hot pressing parameters of UF resin and UF-UPA_6N_ resin adhesives are described in Table 1.

### 2.6. Particleboard Performance Testing

The internal bonding strength (IB), surface bonding strength (SS), thickness swelling rate (TSR), modulus of elasticity (MOE), modulus of rupture (MOR), and formaldehyde emission (FE) of all the particleboard samples were tested for evaluation the modification effect of UF by UPA_6N_. We tested the particleboard performance according to the Chinese GB/T 4897-2015 standard. Each test piece was repeated 6 times, and the average value was taken as the final data.

## 3. Results and Discussion

### 3.1. Structure Analysis of UF Resin and UF-UPA_6N_ Resin

**Figure 3:**
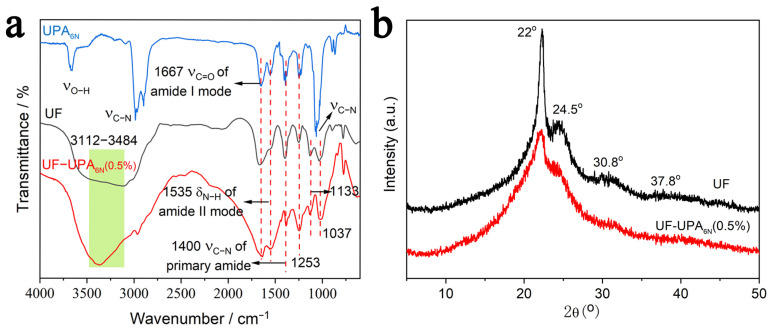
Structure analysis of UPA_6N_, UF resin, and UF-UPA_6N_ resin, FTIR spectra (**a**), and XRD patterns (**b**).

Figure 3a shows FTIR spectra of low molar ratio UF resin, UPA_6N_, and UF-UPA_6N_ (0.5%) resin. As many reports have explained, the chemical and physical properties of UF resin modified with the high-branched structure are quite different from those of low molar ratio UF resin [32,33]. Generally, UF resins with a higher molar ratio have higher free formaldehyde than lower molar ratio UF resins, but the higher molar ratio resins have stronger reactivity and more cross-linking structures than UF resins with a lower molar ratio. Therefore, it is often necessary to modify the structure of UF resin to improve its crosslinking structure. For FTIR spectra of UPA_6N_, characteristic FTIR peaks of the urea linkage, including *ν*(C-H) of CH_2_, *ν*(C=O) at about 1667 cm^−1^ (amide I mode), and δ(N-H) at about 1535 cm^−1^ (amide II mode), are observed in the branched polymer. On the other hand, asymmetric bands of C-O-C and O-C-N oscillations of aliphatic ether are found near 1133 cm^−1^ in UF resin and are stronger than UF-UPA_6N_ (0.5%) resin because most of the ether bonds in UF resin are linear [34,35]. In addition, the FTIR spectra of UF-UPA_6N_ (0.5%) resin shows a broad peak at 3112–3484 cm^−1^, which is ascribed to the *ν*(O-H) of the component. Relative to the FTIR spectrum of UF resin, the (C-N) peak of primary amide (-CO-NH_2_) in the UF-UPA_6N_ (0.5%) resin increases significantly at about 1400 cm^−1^, which is caused by the polycondensation reaction between urea group in UPA_6N_ resin and the amino group in low molar UF resin. This also confirms that the polycondensation reaction does actually occur between the UPA_6N_ polymer and UF resin.

In order to understand the effect of the modifications on UPA_6N_-modified UF resins, the crystal structures of the UF resin and UF-UPA_6N_ resin are measured by XRD. In the case of UF-UPA_6N_ (0.5%), Figure 3b shows the XRD patterns of UF resin and UF-UPA_6N_ (0.5%) resin. From the test results, it is obvious that UF resin has a diffraction pattern of crystallization; these characteristic peaks are observed at 2*θ* = 22.0°, 24.5° and 30.5°, 37.8°. After UPA_6N_ resin is added, the XRD patterns of UF-UPA_6N_ (0.5%) change significantly into an amorphous structure. The accumulation of linear molecules results in highly ordered structures or agglomerations of the low molar ratio UF resin, which are arranged in a manner similar to colloidal particles and peptides. On the other hand, the essential cause of resin crystallization is hydrogen ions (H^+^) generated by an acidic curing agent added before UF resin curing, which extends the length of linear oligomer through a condensation reaction, thus promoting the crystal growth of low molar ratio UF resin [36]. Finally, more N-H and C-O hydrogen bonds are formed, which leads to the formation of an ordered filling structure in the UF resin. In contrast, the structure of UF resin modified by UPA_6N_ is amorphous, indicating that the high branching degree of UPA_6N_ hinders the ordered arrangement of hydrogen bonds in low molar ratio UF resin. More importantly, Park et al. studied the crystallinity of UF resin with high and low molar ratios by using XRD patterns and reached the same conclusion as in this work [6,12].

### 3.2. AFM Images of UF Resin and UPA_6N_-UF Resin

**Figure 4:**
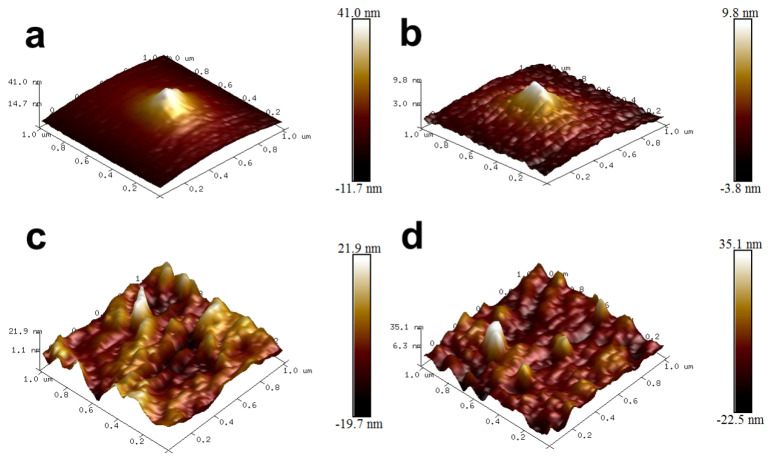
AFM images of UF resin (**a**,**b**) and UF-UPA_6N_ (0.5%) resin (**c**,**d**).

Figure 4 shows AFM images of UF resin and UF-UPA_6N_ (0.5%) resin films. As expected, the images of UF resin and UF-UPA_6N_ (0.5%) resin are significantly different. For example, Figure 4a,b exhibits the UF resin film with a scanning size of 1 × 1 μm. It can be seen from the figure that the microscopic morphology of UF resin film with a low molar ratio presents a layered crystalline structure similar to fish scales because the UF resin is dominated by linear molecules and less branched molecules. During the curing process, the small linear molecule of UF resin has less steric hindrance than the branched structure, making it easier to form a large number of hydrogen bonds between molecular chains, resulting in more crystalline lamellar structures, which is consistent with many reported results [12,23]. The white protrusions in the UF film area may be due to the evaporation of water and formaldehyde during curing. In contrast, the microstructure of UF-UPA_6N_ (0.5%) resin film is rough, showing many bulging and depressed holes (Figure 4c,d). These lumps and holes are due to the three-dimensional network structure formed during the curing process of UF-UPA_6N_ (0.5%) resin film. As mentioned above, UF-UPA_6N_ resin is mainly composed of branched molecules. Therefore, lumps and depressions are formed in some places, resulting in uneven surfaces. In addition, the degree of crystallinity and distribution of dried urea-formaldehyde resin tested by XRD also show similar differences.

**Figure 5:**
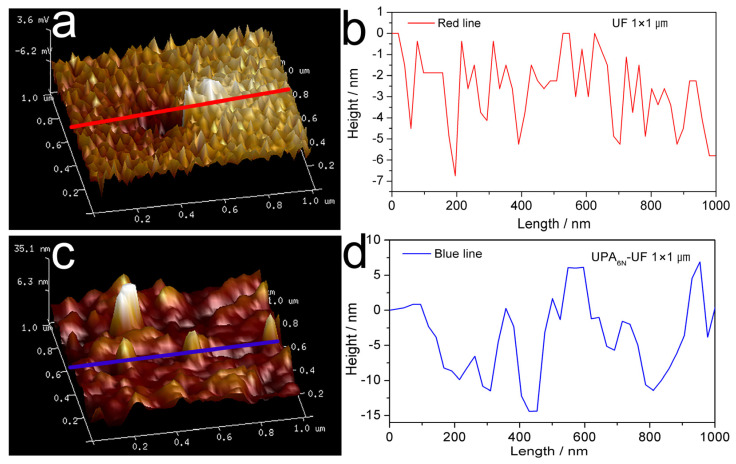
Height profile of AFM images of UF resin film (**a**,**b**) and UF-UPA_6N_ (0.5%) resin film (**c**,**d**).

Figure 5 shows the height profile of AFM images of UF resin film with a low molar ratio and UF-UPA_6N_ (0.5%) resin film obtained by the solid red and blue lines above Figure 5a,c. As can be seen from Figure 5b, there are many small peaks on the red line, about 2–7 nm high, which constitute the layered structure of the crystal, while the three large peaks on the blue line are about 20 nm high (Figure 5d). This is because more dense three-dimensional cross-linking networks are formed in the space of UF-UPA_6N_ resin, and the molecular composition is mainly branch chain, so the plane height of UF-UPA_6N_ resin film is much higher than that of UF resin film with low molar ratio. These results further confirm that UPA_6N_-modified UF resin with a low molar ratio changes its internal structure greatly, thereby effectively improving the mechanical strength and water resistance of UF resin.

### 3.3. TEM Images of UF Resin and UF-UPA_6N_ Resin

**Figure 6:**
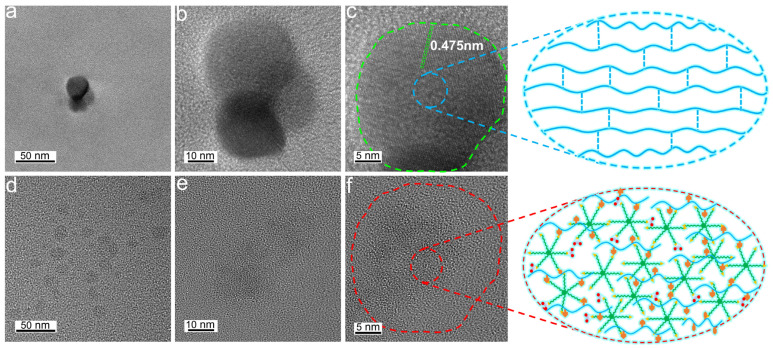
TEM (**a**,**b**) and HRTEM (**c**) images of UF resin films cured at low molar ratio; TEM (**d**,**e**) and HRTEM (**f**) images of UF-UPA_6N_ (0.5%) resin films curing.

The cured morphologies of the UF resin and UF-UPA_6N_ (0.5%) resin are observed by TEM (Figure 6). In this study, UF resin is uniformly coated on a glass plate, and its crystalline morphology is observed after heating and curing. Figure 6a–c show the microstructure of UF resin cured at a low molar ratio. Figure 6d–f show the microstructure of UF-UPA_6N_ (0.5%) resin curing. In addition, at 200 K magnification (Figure 6a), spherical black spots are detected. As mentioned in previous studies, these spherical points are thought to represent spherical/spherical particles in the crystal domain. Figure 6c shows a higher-resolution TEM image with the corresponding chemical structure. Surprisingly, typical layered structures representing individual polymer chains are found at certain locations (e.g., green circles section). The results show that the curing structure of low molar ratio urea-formaldehyde resin is polycrystalline. These lamellar structures most likely form a black spherical dot seen in the previous figure (Figure 6a). Consistent with AFM results, hydrogen bonds are formed between the oxygen and nitrogen hydrogen groups in the linear oligomer of UF resin, resulting in ordered lamellae. As illustrated in Figure 6c, the high-resolution TEM (HRTEM) image of UF resin displays excellent lattice fringes, and the crystal lattice spacing is about 0.475 nm. On the other hand, no ordered crystal structure appears in the enlarged image of UF-UPA_6N_ (0.5%) resin (Figure 6d), indicating that it is mainly composed of irregular amorphous structures (e.g., red circles section). Overall, these results indicate that UF resins with low molar ratios have rich lamellar crystal domains, which represent linear polymer chains interconnected by hydrogen bonds. However, no regular crystal structure is observed in the UF resin modified by UPA_6N_, which is replaced by an irregular branch chain structure, suggesting successful modification of UF resin by UPA_6N_ polymer.

### 3.4. Thermodynamic Analysis of UF Resin and UF-UPA_6N_ Resin

**Figure 7:**
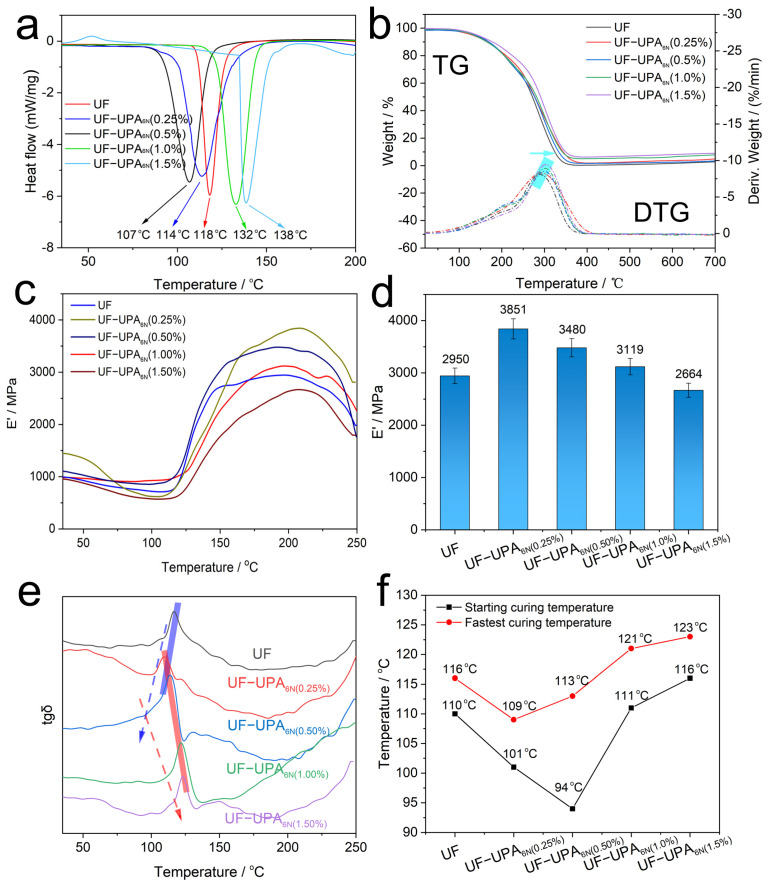
Thermodynamic analysis of UF resin and UF-UPA_6N_ resin. (**a**) DSC and (**b**) TG-DTG curves; (**c**–**d**) E′ value of storage modulus; (**e**) tan *δ* obtained from DMA data; (**f**) the starting and fastest curing temperature.

The curing kinetics of UF resin and UF-UPA_6N_ resin adhesives at a fixed heating rate are evaluated by DSC. As seen from Figure 7a, DSC curves present the curing behavior, and the curing temperature of UF, UF-UPA_6N_ (0.25%), UF-UPA_6N_ (0.5%), UF-UPA_6N_ (1.0%) and UF-UPA_6N_ (1.5%) are 118 °C, 114 °C, 107 °C, 132 °C and 138 °C, respectively. In brief, the curing temperature of hyperbranched UPA_6N_-modified UF resin is relatively low, which can reduce the energy consumption of the production procedure. Therefore, the hot-pressing temperature for subsequent mechanical experiments is set at 200 °C to adjust for the discrepancy in various adhesives. In addition, the thermal stability of adhesive is a key factor in the particleboard production process, and it is very important to assess the performance of the particleboards. TG-DTG test is a typical approach for evaluating the stability and thermal decomposition behavior of polymers. Figure 7b shows the TG-DTG curves for UF resin and UPA_6N_-UF resin. All samples are disintegrated at approximately 150–350 °C; their weight loss for UF-UPA_6N_ resin increases gradually compared with UF resin. *T*_max_ is the temperature at which the weight loss rate reaches its maximum value, and there can be seen that the *T*_max_ of UF-UPA_6N_ resin distinctly increases compared with that of the neat UF resin, which is caused by dense cross-linking structures formed by new covalent bonds between UF and UPA_6N_. These data show that the thermal stability of main components is enhanced with the hyperbranched structural modification of UF resin by UPA_6N_ polymer.

For investigating the temperature effect on modulus, the dynamic mechanical analysis (DMA) is tested by simulating the process of hot pressing and curing of particleboard samples, and storage modulus (E′) and tan *δ* curves of samples are monitored. The elasticity or rigidity of the material can be evaluated by storage modulus [36,37]. The peak temperature of tan *δ* reflects the glass transition temperature of materials to some extent. Figure 7c exhibits E′ curves of UF resin and UF-UPA_6N_ resin adhesives. Relative to UF resin adhesive, E′ values of UF-UPA_6N_ resins adhesives increase with rising the additional amount of UPA_6N_ (from 0 to 1.0%), indicating high storage modulus. However, there is a gradual downward trend in UF-UPA_6N_ resins adhesives (from 0.25 to 1.5%), indicating that the rigidity or elasticity of UF-UPA_6N_ resins is reduced gradually with the increase of the terminal functional group of polyamines (Figure 7c,d). Moreover, values of tan *δ* versus temperature are displayed in Figure 7e,f. Take UF-UPA_6N_ (0.5%) as an example; the E′ value hardly changes when the temperature is lower than 94 °C. Then, the temperature continued to rise, and the E′ value slowly increased and reached the maximum value (3480 MPa) at 113 °C. Next continued to heat up, and the modulus dropped gradually. In a similar way, UF, UF-UPA_6N_ (0.25%), UF-UPA_6N_ (1.0%), and UF-UPA_6N_ (1.5%) increase the maximum modulus at 116 °C, 109 °C, 121 °C and 123 °C, respectively. In total, tan *δ* of UF-UPA_6N_ resin adhesives reduced significantly compared with that of UF resin adhesives, which is attributed to the dense cross-linking structure of UF-UPA_6N_ restricting the movement of chains and reducing the flexibility of materials. By comparison with various mass fractions of UF-UPA_6N_, the UF-UPA_6N_ (0.5%) has a low starting curing temperature and modest fastest curing temperature (Figure 7f). As a result, from the comprehensive analysis of energy dissipation and performance, the UF-UPA_6N_ (0.5%) is an ideal choice for particleboard production.

### 3.5. Physical and the Mechanical Performance Analysis of Particleboard for the UF Resin and UF-UPA_6N_ Resin Adhesives

**Figure 8:**
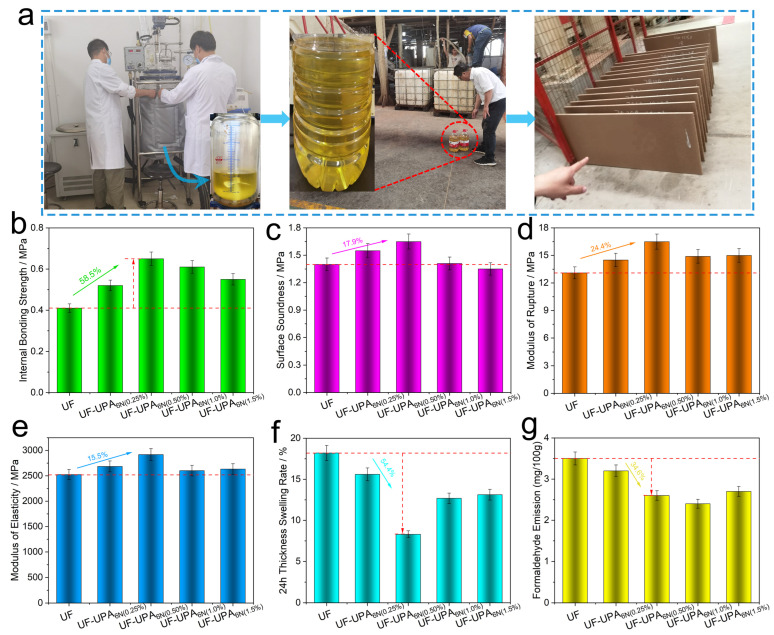
The performance analysis of particleboard for the UF resin and UF-UPA_6N_ resin adhesives. (**a**) The adhesives production process and the prepared particleboard; (**b**) internal bonding strength (IB); (**c**) surface bonding strength (SS); (**d**) modulus of rupture (MOR); (**e**) modulus of elasticity (MOE); (**f**) 24 h thickness swelling rate (24 h TSR); (**g**) formaldehyde emission (FE).

In order to investigate the performance of the modified resin formulation, various mass fractions of UF-UPA_6N_ (from 0 to 1.5% UPA_6N_ content) particleboards are produced on the wood-based panel continuous flat press production line in Yunnan XinZeXing wood-based panel Co., Ltd. Figure 8a shows the adhesives production process and the prepared particleboard. The physical and the mechanical test results of particleboards are illustrated in Figure 8b–g, including the internal bonding strength (IB), surface bonding strength (SS), modulus of rupture (MOR), modulus of elasticity (MOE), 24 h thickness swelling rate (24 h TSR), and formaldehyde emission (FE). As seen in Figure 8b, the IB values of UF, UF-UPA_6N_ (0.25%), UF-UPA_6N_ (0.5%), UF-UPA_6N_ (1.0%), and UF-UPA_6N_ (1.5%) are 0.41, 0.52, 0.66, 0.61 and 0.55 MPa, respectively. With increasing the amount of UPA_6N_ (from 0 to 1.5%), the IB value of UF-UPA_6N_ (0.5%) adhesive increases to the highest 0.66 MPa, which is 58.5% higher than that of the pure UF adhesives. For the SS, MOR, and MOE, the highest values of UF-UPA_6N_ (0.5%) are 1.65, 16.5 and 2917 MPa, which is 17.9%, 24.4, 15.5% higher than that of the pure UF adhesives (Figure 8c–e). Moreover, the 24 h TSR value of UF-UPA_6N_ (0.5%) is the lowest, 8.3%, which is 54.4% lower than that of the neat UF adhesives (Figure 8f).

**Figure 9:**
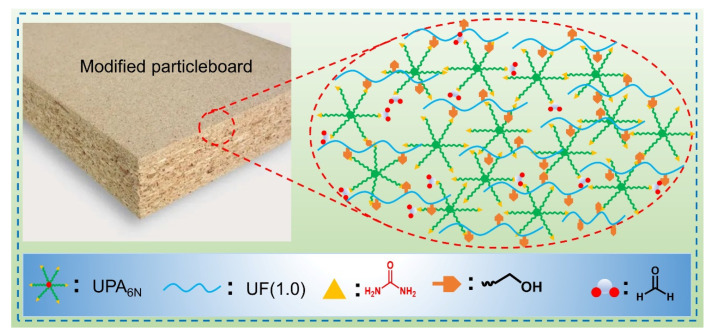
The proposed cross-linked structure mechanism of the UF-UP_6N_ resins used as particleboard adhesives.

For the formaldehyde emission tests, the particleboard bonded with the pristine UF resin resulted in 3.5 mg/100g formaldehyde emission. Figure 8g shows that the addition of UPA_6N_ resin into the UF resin obviously reduced the formaldehyde emission from the particleboard. The lowest FE value of UF-UPA_6N_ (1.0%) is 2.4 mg/100g, which is 45.8% lower than that of the pristine UF adhesives. The UF-UPA_6N_ resins, with the addition of 1.0% UPA_6N_ resins, have more opportunities to reduce formaldehyde emissions. In brief, the UPA_6N_ modified low molar ratio UF resin used as particleboard adhesive exhibits remarkable advancement, especially for the improvement of bonding strength, water resistance, and formaldehyde emission. The mechanism of modification of UF resin by UPA_6N_ may be that the polycondensation reaction between UF resin and UPA_6N_ polymer produces a hyperbranched cross-linked structure, as described in Figure 9. On the other hand, the hyperbranched structure of UF-UP_6N_ adhesives can increase the molecular spacing of the resin, making it easier to swell, thereby improving flexibility and processability [38,39,40]. Meanwhile, excess UF-UP_6N_ can remove the free formaldehyde [41]. This work might provide an excellent strategy for the modification of UF adhesives using branched structures as particleboard adhesives and is expected to achieve industrialization.

In this study, PA_6N_ was added to low molar ratio UF resin as a modifier at 0.25%, 0.5%, 1.0%, and 1.5% by mass, respectively. The results showed that the best effect of resin modification was achieved when the addition of PA_6N_ was 0.5%, after which the performance of particleboard decreased with the increase of PA_6N_ addition. This is because UF resin is cured under weakly acidic conditions, while PA_6N_ resin shows strong alkalinity. When the addition of PA_6N_ resin is small, its effect on the pH of the UF resin itself is not very obvious, and the exposed amine group in PA_6N_ resin can react with free formaldehyde or urea in the UF resin, thus positively promoting the cross-linking curing of the UF resin. As the amount of PA_6N_ resin added increases, its effect on the pH of the UF resin system is greater, and it can even adjust the pH of the UF resin system from weak acidity too strong alkalinity, resulting in the inability of the UF resin to carry out the condensation reaction, thus leading to the performance of the UF resin to drop sharply. The major difference between this study and previous studies is that the mechanism of hyperbranched structure modification of low molar ratio UF resins by PA_6N_ is investigated for the first time at the microstructural level.

## 4. Conclusions

In conclusion, this work focused on the structural modification mechanism of urea-based hyperbranched polymer UPA_6N_ on low molar ratio UF resin and its mechanical properties as a wood adhesive for the preparation of particleboard. The results showed that there were significant differences between UF resin and UF-UPA_6N_ resin in terms of chemical structure, crystallinity, and surface morphology. The UF resin had abundant lamellar crystalline structures, while the UF-UPA_6N_ resin consisted mainly of irregular amorphous structures. In addition, the physical and mechanical properties of UF-UPA_6N_ resin as particleboard adhesive were significantly improved compared with UF resin, with a 58.5% increase in internal bond strength, 24.4% increase in modulus of rupture, 54.4% decrease in 24 h thickness expansion (%), and 34.6% decrease in formaldehyde emission. In short, from a comprehensive analysis of energy dissipation and performance, UF-UPA_6N_ (0.5%) is an ideal choice for the preparation of particleboard. These results indicate that UF-UPA_6N_ resin has great potential to improve the performance of UF resins to obtain high bond strength and low formaldehyde emission. The results demonstrate that the UPA_6N_ modified low molar ratio UF resin (UF-UPA_6N_ resin) used as particleboard adhesives represent remarkable advancement, especially the water resistance has been improved, while the formaldehyde emission has been reduced.

## Figures and Tables

**Table 1:** Hot pressing parameters of UF resin and UF-UPA_6N_ resin adhesives.

Sample	UF (kg)	UPA_6N_ (kg)	Hot Pressing Time (s)	Hot Pressing Temperature (°C)
UF	1000	0.0	180	200
UPA_6N_ (0.25%)	1000	2.5	180	200
UPA_6N_ (0.50%)	1000	5.0	180	200
UPA_6N_ (1.00%)	1000	10.0	180	200
UPA_6N_ (1.50%)	1000	15.0	180	200

## Data Availability

Data will be made available on request.
